# Supplementary figures and images for: Integration of Physiological, Transcriptomic and Metabolomic Reveals Molecular Mechanism of Paraisaria dubia Response to Zn2+ Stress
Source: J Fungi (Basel). 2023 Jun 21;9(7):693. doi: 10.3390/jof9070693 (PMC10381912; doi:10.3390/jof9070693)

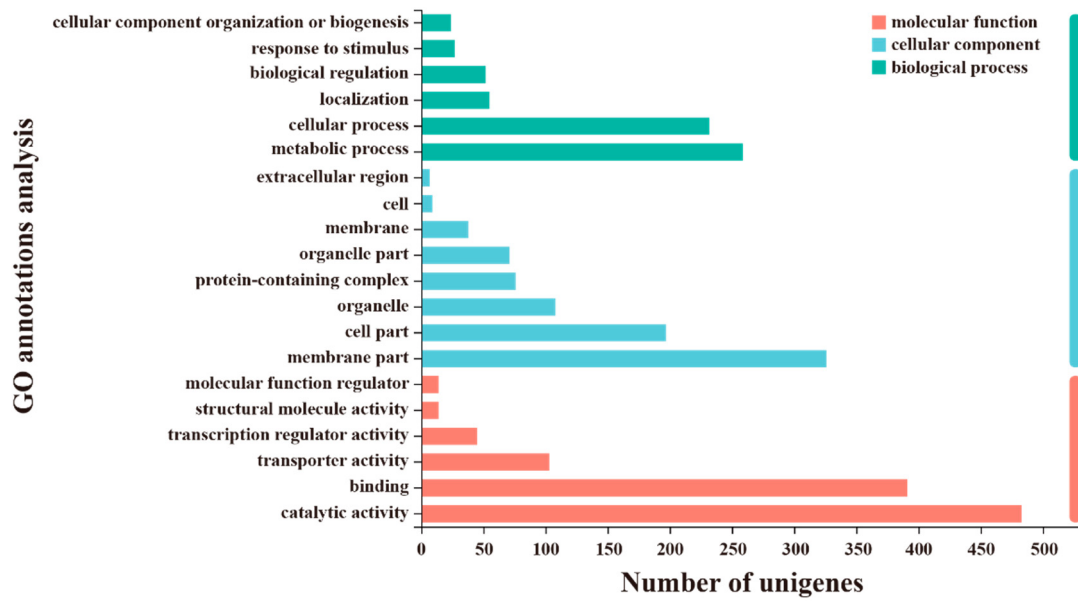

**Figure S1.** Histogram of GO classification of *P.dubia* differential expression genes.

Supplement: Supplementary file 1 [file jof-09-00693-s001.zip › Wang et al. Supplementary data 1.pdf]
